# Targeted CGRP Small Molecule Antagonists for Acute Migraine Therapy

**DOI:** 10.1007/s13311-018-0617-4

**Published:** 2018-03-19

**Authors:** Philip R. Holland, Peter J. Goadsby

**Affiliations:** 1 Headache Group, Basic and Clinical Neuroscience, Institute of Psychiatry, Psychology and Neuroscience, King’s College London, 125 Coldharbour Lane, London, UK; 2 NIHR-Wellcome Trust, King’s Clinical Research Facility, King’s College Hospital, London, UK

**Keywords:** Migraine, Calcitonin gene-related peptide, Gepants, Headache, Neuropeptides

## Abstract

Migraine is a highly prevalent, severe, and disabling neurological condition with a significant unmet need for effective acute therapies. Patients (~50%) are dissatisfied with their currently available therapies. Calcitonin gene-related peptide (CGRP) has emerged as a key neuropeptide involved in the pathophysiology of migraines. As reviewed in this manuscript, a number of small molecule antagonists of the CGRP receptor have been developed for migraine therapy. Incredibly, the majority of the clinical trials conducted have proven positive, demonstrating the importance of this signalling pathway in migraine. Unfortunately, a number of these molecules raised liver toxicity concerns when used daily for as little as 7 days resulting in their discontinuation. Despite the clear safety concerns, clinical trial data suggests that their intermittent use remains a viable and safe alternative, with 2 molecules remaining in clinical development (ubrogepant and rimegepant). Further, these proofs of principle studies identifying CGRP as a viable clinical target have led to the development of several CGRP or CGRP receptor-targeted monoclonal antibodies that continue to show good clinical efficacy.

**Electronic supplementary material:**

The online version of this article (10.1007/s13311-018-0617-4) contains supplementary material, which is available to authorized users.

## Introduction

Migraine is currently ranked the sixth most disabling disorder in the world in terms of disability adjusted life years [1], with ~ 1.04 billion migraine sufferers globally. Given the severe disabling nature of the condition [2], the need for effective acute treatments is clear. Unfortunately, currently available therapies are often nonspecific, poorly tolerated, ineffective, or have cardiovascular contraindications that limit their utility. In fact, 50% of patients report dissatisfaction with current therapies in relation to pain recurrence and almost the same proportion is dissatisfied with the need for supplementary dosing leading to the majority (~80%) of patients considering alternate acute therapies [3]. When we consider the growing concern of medication overuse headache [2] which now ranks in the top 20 disabling disorders [4] globally, the development of novel effective acute therapies is critical. This program of development is borne from an ever increasing understanding of migraine pathophysiology. Migraine is now considered a disorder of the nervous system which is extensively reviewed elsewhere [5–7]. In brief, activation and sensitization of the trigeminovascular system in humans are known to be painful [8, 9] and its stimulation was shown to result in increased levels of calcitonin gene-related peptide (CGRP), pituitary adenylate cyclase-activating peptide (PACAP), and substance P in the cranial circulation of cats and humans [10–13]. A response was later partially confirmed during spontaneous migraines [14]. The trigeminal afferents that arise in the trigeminal ganglion synapse peripherally on the pain sensing intra- and extracranial structures including the dura mater [9, 15, 16] and centrally on the trigeminal nucleus caudalis (TNC) and its cervical counterparts (C1–2). From here, second-order ascending projections terminate in several medullary, brainstem [17–24], hypothalamic [25–30], and thalamic nuclei [23, 29, 31–34]. The trigeminothalamic projections in turn converge on thalamocortical projections that distribute the craniovascular nociceptive signals to multiple cortical regions including the somatosensory, motor, auditory, and visual cortices [35, 36]. Recent studies have highlighted that several of these CNS areas are abnormally active/functionally connected during the earliest attack phases, suggesting that this trigeminovascular activation occurs on a background of dysfunctional sensory integration [7, 37, 38]. Initially, this approach of targeting neuropeptides that were upregulated following experimental trigeminovascular activation and further based on a developing theory of migraine as a disorder of neurogenic inflammation [39] focussed on substance P. Despite initial demonstrations of the ability of substance P antagonists to block this neurogenic inflammation [40], ultimately they failed to translate to the clinic [41], as predicted by a lack of substance P increase in spontaneous attacks [14].

## The Emergence of CGRP as a Therapeutic Target

CGRP belongs to the calcitonin family and is synthesized from either *CALC I* that gives rise to αCGRP or *CALC II* that gives rise to βCGRP [42]. αCGRP predominates in the PNS and CNS, whereas βCGRP is mostly expressed in the enteric nervous system [43]. It is primarily localized to thinly myelinated Aδ and unmyelinated C sensory afferents processing nociception [44]. In fact, ~ 50% of all trigeminal ganglion cell bodies are CGRP immunoreactive [45]. Within the CNS, CGRP is most abundant in the cerebellum [46], with further expression observed in several migraine-relevant brainstem [47, 48], hypothalamic, and thalamic nuclei [49]. With respect to migraine, several lines of evidence point to an important role for CGRP. As discussed previously, circulating levels of CGRP increase in response to trigeminovascular activation or during spontaneous attacks [10, 13, 14]. Given that exogenous CGRP can trigger acute headache and delayed migraine-like attacks in migraineurs [50], it would appear that dampening excessive CGRP signalling may be crucial for attack prevention. In fact, it is known that decreased CGRP release is 1 potential mechanism of action of the 5-HT_1B/D/F_ receptor agonists, the triptans [51, 52].

In agreement with the potential for CGRP targeted therapies for migraine, several small molecule CGRP receptor antagonists were developed with considerable clinical promise [53]. Whereas more recent research has focussed on monoclonal antibodies targeting either CGRP or its receptor (reviewed elsewhere in this special issue) for the prophylactic treatment of migraine [54, 55], the current review will focus on the development of the “gepant” class of compounds for acute migraine therapy.

## Small Molecule CGRP Antagonists

For acute migraine therapy, there has been a total of 6 CGRP receptor antagonists developed (Table 1). These are olcegepant (BIBN4096BS), telcagepant (MK-0974), rimegepant (BMS-927711), BI 44370 TA, MK-3207, and ubrogepant (MK-1602), whereas other molecules such as MK-8031 remain to be explored more fully. In the following section, we will review the clinical trials and selected preclinical research conducted so far for each of these 6 gepants.

**Table 1:** Completed and ongoing clinical trials for gepant compounds discussed in the current review

Small molecule antagonist	Also known as	References	Current developmental stage	Ongoing clinical trials
Olcegepant	BIBN4096BS	[53, 56]	Discontinued	N/A
Telcagepant	MK-0974	[57–63]	Discontinued	N/A
MK-3207		[64]	Discontinued	N/A
Ubrogepant	MK-1602	[65]	Ongoing	[66–68]
BI 44370 TA		[69]	Unknown	
Rimegepant	BMS-927711 BHV3000	[70]	Ongoing	[71–73]

N/A = not available

## Olcegepant (BIBN4096BS)

Olcegepant emerged as the first successful nonpeptide antagonist of the CGRP receptor over 10 years ago [53]. Despite this groundbreaking step, its poor oral bioavailability significantly limited its clinical efficacy and ultimately prevented its progress to further trials. Initial preclinical research highlighted the potential for olcegepant to inhibit vasodilation as a result of trigeminovascular activation or exogenous CGRP [74–76]. However, its anti-migraine efficacy is most likely in response to its ability to modulate trigeminovascular activation. Several early studies suggested a potential central site of action. Olcegepant inhibits TNC activity in response to stimulation of the dural vasculature [77, 78], despite having had no apparent effect on trigeminal ganglion activation [77], whereas its administration direct into the CNS blocks CGRP induced photophobia [79] in receptor activity modifying protein 1 (RAMP1) overexpressing mice and inhibits trigeminovascular nociceptive responses at the level of the TNC [78], periaqueductal gray [80], and thalamus [49]. Despite this clear evidence of a central mechanism of action, the limited brain penetrability of such large molecular weight compounds has resulted in an ongoing debate regarding the peripheral *versus* central sites of action [81].

### Clinical Trials

Initial trials exploring increasing doses of olcegepant (0.1–10 mg) in 55 healthy volunteers [56] highlighted no immediate safety concerns. There was no apparent vasoconstriction, in agreement with previous findings [82] and adverse events were largely limited to the highest doses (5 and 10 mg), mainly transient paresthesias. Follow-up studies on 126 migraineurs explored several doses (0.25–10 mg), with 2.5 mg selected for final analysis. Of the 32 patients receiving this dose 21 reported significant headache relief at 2 h (66%), compared to 11 of 41 (27%) of placebo. In agreement, olcegepant was more effective than placebo for the secondary endpoints of pain free at 2 h, sustained response over 24 h, headache recurrence, and improvement in nausea, photophobia, and phonophobia. Adverse events were in agreement with the phase I study and largely consisted of mild paresthesias (7.3%) with no serious adverse events reported. As noted, the large molecular weight of olcegepant and subsequent reliance on intravenous administration resulted in cessation of its development as new orally available antagonists were sought.

## Telcagepant (MK-0974)

Telcagepant was the first oral CGRP receptor antagonists developed following the initial clinical promise of olcegepant. While telcagepant has undergone several clinical trials, preclinical research is limited with most research utilizing the intravenous utility of olcegepant as noted previously. This is in part likely due to the > 1500-fold lower affinity for telcagepant for the rodent receptor when compared to the human receptor [83]. Notwithstanding this limitation, telcagepant has been shown to inhibit the vasodilatory effects of CGRP on rodent middle cerebral arteries, while having no effect on basal tone, suggesting no vasoconstrictive effect [84]. This is in agreement with clinical trial data in patients with stable coronary artery disease, whereby no significant drug-related cardiovascular adverse events were reported [85]. Additionally, when administered to trigeminal ganglion and smooth muscle cell cultures from human RAMP1 overexpressing mice, with increased CGRP, telcagepant significantly inhibited CGRP induced increases in cAMP production [86]. This effect was not observed in wild-type mice lacking the human RAMP1, further highlighting the limited preclinical utility of telcagepant.

### Clinical Trials

Given the continued debate regarding the potential site of action of small molecule CGRP antagonists such as telcagepant, it is important where possible to address the question of potential central sites of action. Using a blood–brain barrier penetrant PET tracer ([11c]MK-4232) that shows rapid brain uptake and distribution based on the known CGRP receptor expression [87] in the rhesus monkey, Hostetler et al. explored the *in vivo* CGRP receptor occupancy of telcagepant [88], initially demonstrating that a supramaximal dose (1120 mg) of telcagepant decreased CNS receptor occupancy of the PET tracer. However, when administered at clinically relevant doses (140 mg), telcagepant did not significantly reduce the tracer uptake into the brain, suggesting low central telcagepant receptor occupancy.

An initial study exploring the safety and tolerability of telcagepant across multiple doses (25–600 mg) identified no significant adverse events [57]. The most common adverse events observed were nausea, dizziness, and somnolence at the higher doses (300–600 mg). Clinical efficacy was not observed at doses under 300 mg, and as such, these doses were discontinued. Pain relief at 2 h was 68%, 48%, and 67% for the 300, 400, and 600 mg doses, respectively, which was comparable to rizatriptan (10 mg; 69%) and higher than placebo (46%). Secondary endpoints were generally in agreement, with a significant effect of telcagepant compared to placebo, including for pain freedom at 2 h, 24 h pain relief, 24 h sustained pain freedom, and photo/phonophobia freedom at 2 h.

A subsequent phase II trial explored the efficacy and tolerability of telcagepant when combined with nonsteroidal anti-inflammatory drugs (NSAIDS). The dose selected was slightly lower than the 300 mg suggested in the initial study by Ho et al. [57] at 280 mg ± NSAID (ibuprofen 400 mg or acetaminophen 1000 mg) [58]. The primary endpoint of 2 h pain freedom when compared to placebo (10.9%) was met across all groups, ranging from 31.2% for telcagepant alone to 38.3% for telcagepant with acetaminophen. Despite the higher pain-free rates, co-administration of a NSAID had no significant additive effect compared to telcagepant alone. Again, there were no serious adverse events and the most common adverse events were dry mouth, nausea, fatigue, dizziness, somnolence, and tremor. These were more common in the active groups and most commonly reported in those receiving combination therapy [58].

Several phase III studies have further supported the anti-migraine efficacy of telcagepant. Ho et al. [59] conducted a large (1380 patients) multi-center randomized, parallel treatment, placebo-controlled, double-blind, trial comparing telcagepant (150 or 300 mg) with zolmitriptan (5 mg). Both active compounds were well tolerated and side effects were highest for zolmitriptan, lower for telcagepant and lowest for placebo, being similar to those previously reported. Telcagepant at 300 mg met all primary endpoints when compared to placebo, that is, percentage of patients reporting pain freedom, pain relief and absence of photophobia/phonophobia/nausea at 2 h. There was no significant difference between telcagepant and zolmitriptan; however, a follow-up posthoc analysis identified that telcagepant was more effective in those patients who reported as triptan nonresponders or triptan naïve [89]. A second-phase III study confirmed the above findings and further demonstrated that telcagepant at 150 mg was also more effective than placebo [60]. Similarly, a randomized, double-blind, placebo-controlled trial demonstrated a significant effect for telcagepant at 140 and 280 mg across 4 attacks [61]. Patients receiving telcagepant reported increased pain freedom, pain relief and absence of photophobia/phonophobia/nausea at 2 h as well as sustained pain freedom up to 24 h.

The longer-term tolerability of telcagepant (12–18 months) was explored in comparison to rizatriptan [90]. More patients discontinued telcagepant compared to rizatriptan (38.2 and 30.9%, respectively). Both treatments were well-tolerated with dry mouth, nausea, dizziness, and somnolence again appearing as the most common adverse events to CGRP antagonism. Interestingly, the prolonged intermittent use of telcagepant did not appear to impact the liver enzyme aminotransferase, with only transient, asymptomatic, and temporally unrelated (to dosing schedule) elevations observed. This is in direct comparison to studies exploring daily telcagepant for 7 days [62] or 12 weeks (twice daily) [63], which demonstrated increased aminotransferase levels and in the case of the twice-daily regimen, were terminated early, highlighting severe issues regarding daily dosing regimens. These concerns ultimately led to the discontinuation of telcagepant development, despite its clear anti-migraine efficacy and apparent safety when administered intermittently.

### MK-3207

MK-3207 followed on from telcagepant as the second orally available small molecule CGRP receptor antagonist. While there have been limited preclinical studies exploring its efficacy, a labelled version ([^3^H]MK-3207) has been developed to map receptor binding distribution. Initial studies focussed on the cerebellar cortices due to their high expression of CGRP receptors. In rhesus monkey cerebellar slices, high binding was observed in the molecular layer with no binding in the granular layer [91] in agreement with the distribution of CGRP receptors in the molecular layer and in Purkinje cells. The cerebellar distribution and antagonist binding is considered on the background of an increasing understanding of a potential role for the cerebellum in migraine, including its activation during migraine and the earliest premonitory phases [92–94]. More recently, an autoradiographic study using [^3^H]MK-3207 has mapped its widespread binding throughout the rhesus monkey brain slices, including several migraine relevant areas (hypothalamus, periaqueductal gray, dorsal raphe nucleus, and spinal trigeminal nucleus) [95]. Modification of MK-3207 led to the development of MK-8825, which demonstrates increased *in vivo* potency in the rodent, suggesting its utility as a preclinical tool [96]. In a preclinical model of cortical spreading depression (CSD)-induced pain behavior, MK-8825 significantly reduced resultant pain behaviors in rats without blocking CSD itself [97]. Interestingly, this data indicates that CGRP antagonism preferentially impacts on trigeminal nociception, with little effect on CSD, the experimental correlate of migraine aura. In agreement with the ability of MK-8825 to inhibit trigeminovascular nociceptive pathways, MK-8825 was able to block both nitroglycerin-induced increase in activity in spinal trigeminal neurons [98] and nitroglycerin-induced hyperalgesia in rats [99].

### Clinical Trials

A phase II randomized, double-blind, placebo-controlled dose-finding study explored the efficacy of MK-3207 across several doses (5, 10, 20, 50, 100, and 200 mg). Combination of all doses highlighted a positive dose–response trend for pain freedom at 2 h and sustained pain freedom up to 48 h. 2-h pain freedom was significant at all doses above 10 mg when compared to placebo, whereas secondary outcomes of 2 h freedom from photophobia/phonophobia/nausea and 2 to 24 h sustained pain freedom were significant at the 200 mg dose only. Similar to other gepants, the adverse events were most commonly nausea, dizziness, fatigue, dry mouth, and sleepiness [64]. The clinical development of MK-3207 was halted because of hepatotoxicity concerns as previously noted for telcagepant.

### Ubrogepant (MK-1602)

Ubrogepant is an orally available small molecule antagonist of the CGRP receptor that chemically distinct from both telcagepant and MK-3207. A phase IIb, multicentre, randomized, double-blind, placebo-controlled trial [65] explored the efficacy of ubrogepant across several doses (1, 10, 25, 50, and 100 mg). The trial demonstrated a positive dose–response for 2 h pain freedom, and when compared to placebo, the 100 mg dose demonstrated increased pain freedom (25.5% for ubrogepant and 8.9% for placebo). Uncorrected analysis further suggested efficacy of the 50 (21%) and 25 (21.4%) mg doses for 2 h pain free scores. The highest dose further demonstrated efficacy for sustained pain freedom (up to 48 h) and the absence of photo- and phonophobia at 2 h, with no impact on nausea. Adverse events were similar between groups and most commonly included dry mouth, nausea, fatigue, dizziness, and somnolence [65]. Unlike for telcagepant and MK-3207, ubrogepant is currently undergoing phase III clinical trials. Initial studies were due for completion in late 2017–early 2018, with trials exploring the long-term safety and tolerability due to be completed in late 2018 [66–68].

### BI 44370 TA

A single smaller (341 patients) phase II-randomized, double-blind, placebo-controlled trial has been conducted to date to explore the efficacy of BI 44370 TA [69], with patients receiving BI 44370 TA at 50, 200, or 400 mg, eletriptan at 40 mg or placebo. Both BI 44370 TA at 400 mg (27.4%) and eletriptan (34.8%) met the primary endpoint of 2 h pain freedom when compared to placebo (8.6%) and further showed efficacy toward the secondary endpoints of pain relief (56.2 and 56.5% for BI 44370 TA and eletriptan respectively), compared to placebo (18.6%). There was a similar effect for the absence of photophobia, phonophobia, and nausea. Sustained pain-free up to 48 h was only significant for BI 44370 at the 400 mg dose (19.2%) and not for eletriptan (15.9) when compared to placebo (7.1%), while sustained pain relief up to 48 h was significant for BI 44370 TA at both 200 (35.4%) and 400 mg (39.7%) doses as well as eletriptan (34.8%) compared to 11.4% for placebo.

### Rimegepant (BMS-927711)

Rimegepant is 1 of the final small molecule CGRP receptor antagonist that remains in clinical development [71–73] with phase III studies due for completion in early 2018 and a further open label long-term safety study (under the alternate name of BHV3000) currently recruiting and due to complete in early 2019. A single double-blind, randomized, placebo-controlled, dose-ranging trial has been reported [70] exploring rimegepant at 10, 25, 75, 150, 300, and 600 mg or sumatriptan at 100 mg. For the primary endpoint of pain-freedom at 2 h, the maximal effects were observed in the sumatriptan (35%) and rimegepant 150 mg group (32.9%) when compared to placebo (15.3%). Rimegepant at 75 mg (31.4%) and 300 mg (29.7%) also significantly increased pain-freedom at 2 h; however, the higher dose of 600 mg had no significant effect (~25%). For the secondary endpoints of freedom from photophobia and phonophobia, rimegepant at 75, 150, 300, and 600 mg as well as sumatriptan had a significant effect at 2 h that continued up to 24 h. Importantly, given the liver toxicity concerns, 2 patients presented with increased hepatic enzymes as adverse events, 1 in the 75 mg group, and 1 in the placebo group; however, no patients had an increase in aminotransferase levels.

## Discussion

In terms of migraine therapy, CGRP and CGRP receptor-targeted therapies hold significant promise. Building on the initial observations of elevated CGRP in response to trigeminovascular activation in human and preclinical studies as well as during migraine attacks [10, 14], it has become apparent that CGRP release is a key target for migraine therapies, including the established triptan compounds [100, 101]. The seminal study of Olesen et al. [53] demonstrating the efficacy of olcegepant in migraine patients triggered a broad array of studies with several related CGRP small molecule antagonists (Table 1). Remarkably, the majority of studies showed clinical efficacy when compared to placebo highlighting the clear potential of targeting CGRP signalling, that is pain-freedom at 2 h, with further benefits on sustained pain relief out to 48 h and reduction in the presence of associated symptoms such as photophobia, phonophobia, and nausea.

The majority of acute intermittent dosing studies also suggested a reasonable safety profile with minimal serious adverse events, including, dry mouth, nausea, fatigue, dizziness, and somnolence. The therapies were generally well tolerated [90] and the highlighted liver hepatotoxicity concerns surfaced with a transition from their intermittent acute use to a more chronic prophylactic use. On the surface, it may therefore appear that the majority of the gepants were withdrawn from clinical development too early. It should be noted that the triptans suffer from the potential of inducing medication overuse headache in migraine patients when used on more than 10 days per month [102], and as such, there is the potential for overuse of medication in migraine. Given the increased levels of aminotransferase [103] in patients using the small molecule antagonist telcagepant daily for as little as 7 days [62], it is clear that the potential for gepant overuse exists. Thus, despite the apparent safety of acute intermittent dosing, their potential overuse and subsequent potential hepatotoxicity remain as important safety concerns that likely aided in the decision to halt the development of several compounds. Irrespective of the above issues, 2 small molecules remain active in clinical development with ongoing phase III clinical trials for ubrogepant and rimegepant [66–68, 104].

Given the transition toward a novel class of monoclonal antibodies for prophylactic migraine therapy (umabs, reviewed elsewhere in this special issue), and their lack of oral bioavailability, there remains a significant unmet need for novel acute migraine therapies. Recently, the 5-HT_1F_ agonist drug lasmiditan has passed phase III (reviewed elsewhere in this special issue) trials for acute migraine therapy and the successful development of ubrogepant and rimegepant would certainly increase the pharmacological toolbox. Critically for the gepants, despite potential overlapping mechanisms of action with the triptans (modulation of CGRP signalling), the posthoc analysis of data exploring telcagepant and zolmitriptan showed an increased effect of CGRP receptor antagonism in those patients who reported as triptan nonresponders or triptan naïve [89]. Given that the triptan response varies significantly between agents and from patient to patient, with ~30 to 40% of patients not responding adequately to therapy [102], it is clear that there is significant scope for novel acute therapeutic compounds, especially those shown to work in triptan nonresponders.

## Conclusion

Phase I–III trials clearly indicate the potential of small molecule antagonists of the CGRP receptor; indeed, the groundbreaking development of the gepants has ultimately led to the development of multiple monoclonal antibodies targeting either CGRP or its receptor. The majority of studies have produced positive efficacy results with limited adverse events. It is perhaps unfortunate that their chronic prophylactic use was tested as on the surface it appears that their intermittent use remains safe and efficacious. Notwithstanding the ongoing development of ubrogepant and rimegepant that remains viable candidates for acute migraine therapy, the gepants have enthused fresh life into the migraine field. As we strive for a greater understanding of the mechanisms and potential sites of action of targeted CGRP modulators, this can only help to further advance our mechanistic understanding of this complex highly disabling condition.

## Electronic Supplementary Material

ESM 1 (PDF 1225 kb)
